# Adolescents' Perceptions About Dating and Sexual Permissiveness in Ebonyi State, Nigeria: What Can Be Done to Enhance Adolescents' Sexual Health and Well-Being

**DOI:** 10.3389/frph.2021.626931

**Published:** 2021-07-08

**Authors:** Nkoli Ezumah, Ifunanya Clara Agu, Chinyere Okeke, Chibuike Agu, Chinyere Ojiugo Mbachu, Obinna Onwujekwe

**Affiliations:** ^1^Health Policy Research Group, University of Nigeria, Enugu, Nigeria; ^2^Department of Community Medicine, University of Nigeria, Enugu, Nigeria; ^3^Department of Health Administration and Management, University of Nigeria, Enugu, Nigeria

**Keywords:** adolescents, perceptions, sexual permissiveness, sexual and reproductive health, Nigeria

## Abstract

**Introduction:** Adolescent sexual and reproductive health (SRH) issues constitute key health concerns as some adolescents are directly or indirectly involved in sexual engagements, with increased risks and health consequences. The study aims to explore adolescents' perceptions about dating and permissive sexual behaviors which will contribute to designing sexual and reproductive health interventions. This paper adds to knowledge on adolescents' perceptions about dating, pre-marital, casual, transactional and age-disparate sex in southeastern, Nigeria.

**Methods:** A qualitative study was undertaken in the three senatorial zones of Ebonyi state, south eastern Nigeria. The study population comprised unmarried in- and out-of-school adolescents aged 13–18 years. Data were collected using a pre-tested focus group discussion (FGD) guide. There were six FGDs for boys and six FGDs for girls. A thematic framework approach was used for data analysis.

**Results:** Adolescents' views about dating and other sexual behaviors were varied. The dominant view is that hugging, touching and kissing are inappropriate for unmarried adolescents. Similarly, pre-marital, casual, transactional, and age-disparate sex were viewed as unacceptable. However, some adolescents perceived pre-marital abstinence as a hindrance to the attainment of sexual satisfaction and reproductive capacity in marriage. Some boys and girls indicated that casual sex is good, because it enables girls from poor homes to socialize with more privileged boys/men, and that such relationships could lead to marriage. Some considered transactional and age-disparate sex as a means of survival from poverty and unemployment. Boys were more permissive in their views about sexual behaviors compared to the girls.

**Conclusion:** Adolescents' perceptions of sexual behaviors as acceptable/unacceptable vary and are gendered. This should be considered in designing innovative strategies to improve adolescents' sexual health and well-being.

## Introduction

Adolescent sexual and reproductive health issues constitute key health concerns all over the world (1). Adolescence is a transitional period from childhood to adulthood, which is often associated with heightened curiosity and risk-taking, including sexual risks (2). Cognitions related to risky sexual relationships have been linked to the observed high rates of pre-marital sex and early sexual debut among adolescents, as well as increasing rates of risky sexual practices such as multiple sexual partnering, unprotected sexual intercourse, casual sex, transactional sex and age-disparate sexual relationships (3–7). These risky sexual behaviors have been linked to increased rates of sexually transmitted infections (STIs) including HIV/AIDS, and psychosocial sequel (8). From the Theory of Planned Behavior perspective, sexual intention is determined by a permissive attitude, and the perception of social norms and perceived self-efficacy in performing a sexual activity. Hence permissive attitudes toward sexual activity play an important role in sexual behaviors (9). Sexual permissiveness has been used to define liberal, lax and non-judgmental attitudes toward sexual behaviors including pre-marital sex, cohabitation, casual sex, multiple sexual partnering and age-disparate sexual relationships (10, 11).

A review of age-disparate and transactional sexual relations in sub-Saharan Africa described engaging in sexual relations with older partners as the norm for adolescent girls (8). In Tanzania, young girls and young women engage in transactional sex due to the tolerance of that sexual behavior in that society where men generally are expected to make material provisions for females they have a sexual relationship with and also due to peer pressure. Indeed it was reported that involvement of adolescent girls and young women in transactional sex is socially accepted as long as gifts/material benefits are provided. Hence to avoid labeling or ridicule, adolescent girls and young women engage in transactional sex without bothering about the consequences of the practice on their well-being (9).

The recent Nigerian demographic and health survey (12) report shows that 0.9% of adolescent boys reported ever having paid for sexual intercourse. Furthermore, 7.9% of boys and 9.6% of girls aged 15–19 years had sexual intercourse with persons who were neither their husbands nor lived with them, in the past 12 months preceding the survey (12). Data on the incidence and prevalence of STIs in Nigeria are limited because of the under-reporting of STIs, especially among young persons. However, HIV prevalence among adolescents aged 15–19 is estimated to be 2.9% (12, 13).

In the traditional Nigerian society, strict moral principles, culture, religious and social influences were regarded as restrictions to “socially and culturally unacceptable” sexual behaviors (13–16). For instance, pre-marital sex was frowned upon, sex which was considered a sensitive issue was rarely discussed in the open, and punishments were meted to those who violated such established moral codes in many Nigerian settings. These restrictions and consequences created a culture of silence and prevented open discussion of sexuality between adolescents and well-informed adults (17, 18).

However, it has been reported that cultures that are against non-marital sex are counterbalanced by permissive attitudes reflected in the media and the values of many adults around the adolescents (19). In particular, the increasing exposure to the internet and social media, and consequential adoption of foreign ways of life by young people, have paved the way for more tolerance for (and indeed acceptance of) sexual lifestyles that were previously regarded as offensive or socially unacceptable (14, 20, 21).

Furthermore, evidence shows that factors such as gender and family structure influence adolescents' views and attitudes about sexual behaviors (22, 23). Families with single parents have been associated with permissive parental attitudes and reduced parental control which in turn, influence their adolescents' sexual behaviors (24, 25). On the other hand, family structure and family support are protective factors against sexual behaviors such as transactional sex among adolescents and young adults (17). Studies indicate that males are more likely to initiate sexual intercourse and have more permissive perceptions about sex than females (26, 27). Some motives that encourage permissive sexual activities among adolescents include the desire by males for sexual gratification and the desire by females to develop romantic relationships that will lead to marriage (8, 16); the belief that pre-marital virginity is no longer important; and the notion that sexual freedom is accepted as normal by the contemporary society (28, 29). Other factors associated with liberal and permissive attitudes to sex among adolescents are urbanization, negative peer influence and poor socioeconomic status (5, 16, 30).

The adverse sexual practices such as casual, transactional and age-disparate sexual relationships have been linked to increased risk of unsafe sex and STIs (31–34). Consequently, unwanted (teenage) pregnancies and unsafe abortions resulting from complications of sexual activities among youths, aged 13–21 years old are of public health challenges, especially, in sub-Saharan Africa (35). These can also, lead to psychosocial challenges such as feelings of anger, fear, anxiety, depression, low self-esteem, guilt, stigmatization, isolation, possible drop out from school; economic adversity and even death (3, 14).

Given the increasing impact of foreign influences and changing moral codes on young people's perceptions of what constitutes “socially acceptable” sexual behavior, there is a need to design sexual and reproductive health interventions that aim to address potentially harmful notions about sexuality. An in-depth exploration of permissive sexual behaviors among adolescents will contribute to designing appropriate interventions and strategies.

Other studies in this area have either focused on in-school adolescents or pre-marital sex (14–16, 20, 26, 36–38). Focusing on in-school adolescents alone systematically excludes the views of the many adolescents who are not enrolled in formal education settings. This one-sided view implies that interventions that are designed and/or implemented to address the SRH needs of adolescents may not be comprehensive enough to effectively address the needs of the out-of-school adolescents whose views have been sidelined. Thus, it is imperative that a study that is more encompassing is undertaken to get all shades of opinions.

The study aim to explore adolescents' perceptions about dating, pre-marital, casual, transactional and age-disparate sexual intercourse in Ebonyi State, south eastern, Nigeria. This paper provides new knowledge on the perceptions of in-school and out-of-school unmarried adolescents about sexual permissiveness in Nigeria. The findings will be invaluable to policymakers and SRH program officers in developing intervention strategies that will ensure access to right SRH information and debunk misconceptions to improve adolescents' sexual health and well-being.

## Materials and Methods

### Study Area and Design

This cross-sectional qualitative study was conducted in Ebonyi State, Southeast Nigeria. The state has three senatorial zones, 13 local government areas (LGAs), and 5,533 km^2^ estimated land area. Over 40% of its total population is under the age of 15 years (39) and it is estimated that the population of adolescents will increase in few years as the state records the highest fertility rate of 5.4% among the south-eastern states in Nigeria (12). The maternal mortality rate among girls aged 15–19 years is 39.7 and 8.2 % of girls in this age group in the state have already begun childbearing (12).

### Study Population and Sampling

The study population comprised unmarried in- and out-of-school adolescent boys and girls aged 13–18 years in urban and rural communities of Ebonyi State, Nigeria. Six LGAs were purposively selected to represent the geographical locations (urban and rural) and the three senatorial zones in Ebonyi state. Two LGAs from the list of LGAs that were prioritized by the Ebonyi State government for adolescent sexual and reproductive health (SRH) intervention were selected from each of the senatorial zones. A community (that is a geographic area governed by a traditional ruler) was selected from each of the LGAs during the stakeholders' engagement workshop. In each study community, in-school adolescent boys and girls were randomly selected from public secondary schools while out-of-school boys and girls were randomly selected from their skill acquisition or workplaces. The socio-demographic distribution of focus group discussion (FGD) participants can be seen in Table 1.

**Table 1:** Socio-demographic distribution of focus group discussion (FGD) participants.

**Variables**	**Number of participants**	**Gender**	
		**Female**	**Male**
**Place of residence**			
•Urban	63	32	31
•Rural	71	36	35
**Age category**			
•13–14	32	20	12
•15–16	53	27	26
•17–18	49	21	28
**Schooling (junior and senior secondary)**			
•In-school	96	42	54
•Out-of-school	34	26	12
**Employment status for out-of-school**			
•Employed	29	17	12

### Data Collection

The data were collected using a pre-tested focus group discussion (FGD) guide that was developed based on the study objectives by a team of qualitative research study experts. The FGD guide was pre-tested among adolescents in urban and rural communities in a contingent state. The guide was then reviewed by the team of experts after the pre-testing exercise to ensure sufficient coverage of themes. Before the project commencement, a relationship was established with some of the stakeholders in Ebonyi state during a stakeholders' engagement meeting and community mobilization. We used FGDs in discussions with adolescents because the tool offers two advantages. It provides respondents' perspectives and lived experiences concerning the themes and enables us to identify areas of consensus, and divergences among groups of respondents on the views expressed. The FGDs were conducted for 1 month by experienced qualitative researchers whose capacities were further strengthened through a training workshop facilitated by the primary author. The FGDs were disaggregated by sex, and a total of 12 FGDs were conducted such that six FGDs were held with boys and another with girls. The participants for each FGD ranged from 8 to 13 in number, and the FGDs were scheduled in their most convenient venues to ensure confidentiality. All the participants were informed of the objectives of the study before the interview, self-introduction was carried out and permission to audio record the interviews were obtained. Also, verbal and written consents were obtained from the participants. Each interview was conducted by a moderator and a note-taker. The interviews were conducted in the English language but where necessary, the questions were translated into Igbo for better understanding. The participants were allowed to express themselves in their most preferred language. The FGD interview questions are provided in a Supplementary Material.

### Data Analysis

All the audio files for the focus group discussion were transcribed verbatim in the language that the interviews were undertaken and later translated into English where necessary. A thematic framework approach was used to analyze data. The transcripts were processed, edited and anonymously coded using Microsoft Word. All the data were kept in a password-protected laptop. At the initial stage, all transcripts were read to gain full insight into the data then, the richest transcript was selected for a comprehensive study and coding. The initial or draft key themes and sub-themes relating to adolescents' perceptions about sexual permissiveness were generated and this formed the first coding framework. The coding framework was tested on another rich transcript which was revised and refined to form the final coding framework. The final coding framework was then applied manually to all the transcripts including the two transcripts that were used in generating and testing the framework. The thematic areas in the final coding framework include adolescents' views about and permissiveness for dating (including hugging, touching and kissing); pre-marital sex; causal sex; transactional sex; and age-disparate sex. Data on each of the five themes were recorded in different sections of the transcripts and coded separately to ensure there was no overlap among them. Validation of synthesized data was done immediately after the analysis through a stakeholders' workshop in Ebonyi state.

### Ethical Considerations

The research study protocol was submitted to two Ethics Committees namely; (1) the Health Research Ethics Committee of the University of Nigeria Teaching Hospital Enugu and (2) the Research and Ethics Committee of Ebonyi State Ministry of Health. Before entry into the study site, ethical approval was secured from the two committees. Written informed consent was obtained from parents/guardians of adolescents who participated in focus group discussions. Before data collection participants were informed of the study objectives, benefits and risks of participation, their rights to voluntary participation and confidentiality of data. Written consent was then obtained from study participants.

## Results

The findings on adolescents' views and permissiveness for dating and sexual behaviors are presented under five themes namely: dating (hugging, touching and kissing); pre-marital sex; casual sex; transactional sex; and age-disparate sex. Adolescents' views about tolerance or non-tolerance of dating and other sexual behaviors were divergent and mostly gendered.

### Dating (Hugging, Touching, and Kissing)

Adolescents' views about dating were varied. However, the dominant view expressed by both male and female participants is that dating (hugging, touching, and kissing) is inappropriate and unacceptable for adolescents. Specific reasons provided for non-acceptance of dating are; that dating (hugging, touching, and kissing) is for people who are ready for marriage, it is immoral and pre-disposes people to pre-marital sex which leads to loss of virginity. They further described that engagement in pre-marital sex exposes people to the risk of contracting sexually transmitted diseases, unwanted pregnancy and abortion; dropping out of school by pregnant girls and abandonment by the boy responsible for the pregnancy. Some of the respondents also indicated that kissing is not acceptable because of their perception that it is not proper to swallow another person's saliva. Illustrative quotes to buttress these responses from some male and female participants follow:

*Dating at this age (adolescence) is not advisable*… (R5, ADOHM, male adolescent).

*Boys and girls who are up to puberty age kissing and hugging will make them feel like having sex immediately because they will feel a sexual urge into their bodies. So the concern for sex will make both of them not achieve what they want to achieve in life* (R11, ADEZM, male adolescent).

*Dating is not good because sometimes, the guy might have the urge for sex which leads to sexual intercourse* (R9, ADOHF, female adolescent).

…*could lead to sex, if she is not careful the girl will be impregnated'* (R2, ADIKF, female adolescent).

*Touching and kissing is not good because it is not good to be swallowing another person's saliva* (R1, ADABF, female adolescent).

Concerning tolerance of dating, only male respondents residing in urban communities indicated that dating (hugging, touching and kissing) is acceptable, and a means of expressing natural feelings of love as is obtainable in foreign countries.

*Dating and kissing should be allowed because the feeling is natural and it was God made*… *Kissing and hugging are symbols of greetings and love… So many people reject kissing but in foreign countries kissing is a good thing and in the bible, it was said to greet one another with a kiss of love although African culture said it is bad especially in Nigeria* (R7, ADAFM, male adolescent).

### Pre-marital Sex

Similar to perceptions about dating, the dominant view among adolescents was that pre-marital sex is culturally unacceptable. It was perceived as an immoral behavior because pre-marital virginity is the ideal, and the norm is that only married people should engage in sex. Some mentioned that Christians also regard pre-marital sex as an immoral act. Some respondents thought that it is due to a lack of parental control that some adolescents engage in pre-marital sex. Many also agreed that pre-marital virginity is important for boys and girls to avoid contracting STIs. There were no gender and place of residence differences in adolescents' perceptions about pre-marital sexual engagement.

Responses buttressing these expressions follow:

*What I think is that having sex before marriage is not a good thing. It is an abomination before God and man because having sex is meant for married men and women, not for adolescents* (R1, ADEZM, male adolescent).

…*having sex outside marriage, the Bible regards it as a sin, because sex outside marriage is a sin. You don't have to have sex outside marriage, you have to go into marriage before knowing what sex is all about* (R11, ADEZF female adolescent)

.*Sex is meant for mature boys and girls* (married people) (R4, ADOHM, male adolescent).

…*it is not good to have sex before marriage because you will lose your virginity* (R2, ADOHM, male adolescent).

*When a man and a woman have sex before marriage… the pride of the woman has already been lost. The man will see her as a second hand or “Belgium” (used material) or something like that* (R5, ADEZM, male adolescent).

*What I know is that it is so illegal it is not advisable because it leads to the destruction of the future. After all, if the person eventually gets pregnant she no longer continues with her education* (R3, ADIKF, female adolescent).

Consequences of pre-marital sex outlined by respondents include loss of virginity, risk of contracting sexually transmitted infections, unwanted pregnancy, dumping of the girl by the male responsible when pregnancy occurs, dropping out of school by the pregnant girl, the possibility of the pregnant girl seeking an abortion, and academic distraction for adolescents and disgrace for family members. Illustrative quotes are provided below.

*What I think about boys and girls having sex before marriage is that if you start having sex with a boy and you are not married to him the boy may get you pregnant and dump you* (R4, ADEZF, female adolescent).

*Mistakenly the person will be impregnated and the person will drop out of school* (R10, ADIKF, female adolescent).

*It (pre-marital sex) is bad because it leads to future destruction* (R10, ADEZF, female adolescent).

…*It (pre-marital sex) leads to the destruction of the future because if the person eventually gets pregnant she no longer continues with her education* (R3, ADIKF, female adolescent).

*I think they have not known what they are in that school for, because that sex will make them not be punctual and they will not be able to concentrate on any lesson going on*. (R4, ADEZM, male adolescent).

*I think they are destroying their family image. When a person catches them inside an uncompleted building, the person might announce their name that these persons are doing this sort of evil, from there, it may disgrace their family* (R11, ADEZM, male respondent).

Support for pre-marital sex was expressed mainly by some of the male respondents. Their views are that pre-marital sex resulting in pregnancy enables a prospective husband to ascertain that the girl is fecund. In particular, it was reported that among some communities in the other Nigerian States, pre-marital sex is culturally acceptable and a girl is expected to have a child before marriage to ensure that she is fecund. The following are supportive quotes.

*What I think is that before the marriage will hold, they need to have a child* (R2, ADIKM, male respondent)

…*some people used to say that it is good to have sex before marriage because after the wedding they might not have children*. (R3, ADEZM, male adolescent).

Other reasons provided mostly by male respondents for tolerance of pre-marital sex stem from the misconception that pre-marital sex expands the cervix so that child delivery will not be difficult. The respondents further described that without experience of pre-marital sex, one will not enjoy sex when married and will experience pains during sexual intercourse. Also, that a person who did not engage in pre-marital sex will not know how to have sex in marriage and that if boys do not have pre-marital sex when they get older they will experience reproductive problems. According to some male respondents;

*It is good to have sexual intercourse especially the girls. It helps the girl's cervix to expand and it prevents difficulty in childbirth* (R9, ADAFM, male adolescent).

*What I think is that, if they grow without having sex, when they marry they will not know how to have sex with each other* (R5, ADIKM, male adolescent)

*If you don't want to have sex when you are not married, when you are married there will be a pain when having sex (R6, ADIKM, male adolescent)*

### Casual Sex

The occurrence of casual sex among adolescents was reported in the study, as well as peer pressure to participate in casual sex. Boys cajole and stigmatize other boys who do not engage in casual sex. Some male respondents pointed out that some girls also ridicule boys that do not engage in casual sex with them. But there were mixed responses in respondents' perceptions about the practice. While some adolescents believed that casual sex is bad, others thought that it is acceptable. Respondents' reasons for opposing casual sex include their perception that those who engage in casual sex are under the illusion that sexual intercourse will provide happiness for them whereas the outcomes are risks of contracting sexually transmitted infections and unwanted pregnancy; the pregnant girl may not be sure who is responsible for the pregnancy; the pregnant girl has the risk of dropping out of school; and that adolescents engaging in casual sex are jeopardizing their future.

Supporting quotes are indicated.

“*They think that when they have sexual intercourse they will be happy and it is alright…The answer is it is not ok because it is not only sex that you can use to prove to a person that you love him or her*” (R11, ADIKM, male adolescent).

*They can easily be infected with any disease* (R8, ADEZF, female adolescent)

*It is bad because you don't know if the person has HIV and you can get it from there* (R8, ADOHM, male adolescent)

“*Mistakenly the person will be impregnated and the person will drop out of school*” (R10, ADIKF, female adolescent)

“*Again the female might get pregnant and you won't know the person responsible*"(R9, ADOHM, male adolescent).

*I feel bad about them (peers). This time around they (peers) will say that you are a “Jew man.” That you don't know what is happening, that you are ignorant. They would also say that you are not a man* (R11, ADEZM, male adolescent).

*Some girls that are playing away match (having sex with someone that is not her boyfriend)will say that this guy cannot do anything to her, not since they are friends he has not even had sex with me* (R2, ADEZM, male adolescent).

Concerning support of casual sex, mainly male respondents who are living in urban communities expressed the view that casual sex is good, and could lead to marriage or boyfriend/girlfriend relationships. In particular, it was reported that casual sex enables girls from poor homes to meet, socialize and enjoy with other adolescents. “*One-night stand will make them meet different faces, for those from the poor background they enjoy themselves (eating and drinking)”* (R8, ADAFM, male adolescent). Some of the discussants indicated that peers, friends or schoolmates approved of casual sex, and pressurized others to participate in the practice. As some respondents indicated;

*Yes, this time around they approve it. The way they approve it is that sometimes the girls will put on the type of thing they will put on, that would make the male counterparts to be seduced instantly which will make them either during free time or after lesson, might plan where they might meet to have short-time (casual sex)* (R4, ADEZM, male adolescent).

*Yes, these days you can't say that this boy is not like that, because when school closes, dismisses you have gone home, you don't know about your friend. Maybe on their way coming back home, they might see their girlfriends, their girlfriends may ask them to escort them home. When you escort the girl home, she may ask you to have a seat. She might go inside and take her bath, after the bath the girl might tie a towel on her waist, when she reaches the place that you are sitting, you may not even control yourself again from what your eyes saw* (R11, ADEZM, male adolescent).

### Transactional Sex

While the majority of discussants viewed transactional sex as unacceptable, some male and female respondents regarded it as acceptable in certain circumstances. In particular, adolescents' participation in transactional sex was perceived to occur on account of poverty and lack of employment, and because some have no parents to take care of them. Consequently, some girls resort to exchange sex for material/ financial benefits as a means of supporting themselves. As some respondents opined:

*The reason is that some come from a poor background, they can't afford to feed themselves* (R1, ADEZM, male adolescent).

“*When you don't have anything, no parents to support you, it is acceptable to go for transactional sex, you can go and use your body to get what you want*” (R12, ADOHF, female adolescent)

“*Most times adolescents are not to be blamed because it is the government's fault due to unemployment*…” (R7, ADMFM, male adolescent)

Discussants who were less tolerant of transactional sex thought that adolescents engage in transactional sex due to greed, peer influence, and lack of self-control. Indeed suggested options some provided are that adolescents should be patient, and try to be hardworking and seek alternative means of making ends meet rather than engaging in transactional sex.

Illustrative quotes follow:

…*it is because of greed* (R4, ADIKF, female adolescent).

*Some are caused by the girl. Because when a girl has a longer throat, (is greedy) you won't even know the man, he will call you and you will come. If he asks you your name and you are telling him, the man will give you money, next day he will give you again, another he will ask you to have sex with him and you will not even say no, because you have seen money you will involve yourself in sexual intercourse* (R11, ADEZM, male adolescent).

*I think it is a peer group influence. Because when a girl sees her age mates wearing some clothes, she might think that she knows where they used to get such sources of money. They will now tell her what it takes them to get such things, and enduring such economic condition that is rampant everywhere now, she may kind of say that, she should just join them because they feel that it is something you can just do for few minutes and get your cash. So, mostly what causes that is peer group influence* (R4, ADEZM, male adolescent).

*My take is that it is not good, although some of them are doing it because they don't have parents that is not the reason why they should engage in that instead, they will look for a sponsor, although a sponsor would be demanding something or favor* (R3, ADEZM, male adolescent).

*I think it is not good …The reason why they give you the money is that if they give you the money and you use it to do your hair they will try to take it back from you by using you* (R6, ADEZF, female adolescent).

### Age-Disparate Sex

The dominant view among male discussants was that boys engaging in sex with older women, or girls engaging in sex with older men is immoral, unacceptable and an abomination. Some argued that adolescents are involved in that form of sexual relationship for financial reasons on account of greed and lack of self-control and due to selfishness of the older men or older women. Some regard it as a form of prostitution.

*It is an abomination and should not be heard amongst adolescents* (R6, ADOHM, male adolescent).

*By not being contented with materials being supplied by their parents* (R3, ADIKM, male adolescent).

*Lack of self-control*. (R4, ADIKM, male adolescent).

*Some women can say that they like the lineage of that family. They will be trying to seduce any young boy from that family to have sex with them so that they will have a child with the genotype of that family so that …they will have somebody that looks like that very family* (R4, ADEZM, male adolescent).

Consequences identified that could arise from adolescents' involvement in age-disparate sex include unwanted pregnancy and abortion; and the risk of the older sex partners using the adolescents for ritual activities. There were also misconceptions that age-disparate sex has adverse effects on the physical appearance of the girl. The notion is that while the old man will be refreshed, the young girl will look older than her age because of the assumption that the older man's blood will be transferred to the girl. These responses are reflected in the following quotes:

*You see the kind of days we are in… poverty, so people usually say that they don't have money, a girl may come and say after all this man has money and she will go and meet him, the man can promise her anything she wants. Starting from then she will say after all I come from a poor home and the girl will say ok, I have agreed. And from that, if the man has sex with her and she gets pregnant, the man will say after all we are going to abort the baby* (R9, ADEZM, male adolescent).

*It is not good because when you have sex with older men…It will make you look older than your age* (R1, ADABF, female adolescent).

*It is not good for a young girl to have sex with an old man because when you are having sex with an old man, the man will use your blood to refresh himself. You will be getting old; the man will be getting younger* (R11, ADEZM, male adolescent).

*It is not good for adolescents to engage in sex with older men or women because some of them use adolescents for rituals* (R7, ADAFM, male adolescent).

The reasons some male adolescents provided for their acceptance of age-disparate sex are that girls engage in sex with older men partly due to poverty and that older women seek sexual satisfaction with adolescent boys to achieve pregnancy. According to a male respondent;

*Some women can say that they like the lineage of that family. They will be trying to seduce any young boy from that family to have sex with them so that they will have some type of gene of that family so that …they will have somebody that looks like that very family* (R4, ADEZM, male adolescent).

## Discussion

Adolescents' views about tolerance or non-tolerance of dating and other sexual behaviors were divergent and gendered. The predominant view about dating (hugging, touching and kissing) among adolescents is that it is inappropriate and unacceptable for adolescents. They specifically described the act as immoral, pre-disposing people to having pre-marital sex and risk of contracting sexually transmitted infections, unwanted pregnancy and unsafe abortion among others. Although there were predominant views, gendered divergences in views occurred among adolescents as more girls thought that dating is unacceptable whereas more boys than girls thought that dating is acceptable. In this study, most adolescents indicated that pre-marital sex is unacceptable due to cultural norms and religious values with an emphasis on virginity which they perceive as the pride of a woman that will make a husband proud of his wife. Also, to avoid contracting STIs, unwanted pregnancy and related problems, the adolescents consider pre-marital sex unacceptable. The non-acceptance of pre-marital sex in our study corroborates the findings of Envuladu et al., in Plateau State, northern Nigeria (40). They reported that the reasons provided by adolescents for not having sex are religious beliefs, to avoid STIs and for girls to maintain their virginity which they expect will make their potential husbands respect them in future.

These findings which reflect on cultural and religious beliefs are contrary to the study by Eze, in Anambra state, south east Nigeria, which reported that male and female adolescents indicated they like intimate and casual kissing and breast fondling (16). The study reported that both boys and girls were of the view that there is nothing wrong with having pre-marital sex or having many sexual partners (16). Peer-orientated adolescents were found to be more likely to indulge in kissing, embracing and breast fondling compared to parent-oriented ones (16). Our findings also appeared to vary from what has been reported in studies from the south western part of the country where the majority of the adolescents surveyed were comfortable with dating and a good number had engaged in various forms of sexual activities with more residing in the rural than urban areas (36, 41, 42). These findings were reported by peer-oriented and parent-oriented adolescents who were both students and out-of-school.

The variance of our findings from similar studies could be explained by the difference in the methods of data collection, and the potential for desirability bias to be more evident in focus group discussions when compared to one-on-one interviews. This study utilized focus group discussions whereas the other studies used one-on-one interviews. The implication is that in a group discussion (FGD), there is the likelihood for some participants who are not “bold” or assertive to give responses they feel are socially desirable to sensitive issues in conformity to their cultural and religious views. For example, “most respondents indicated that pre-marital sex is unacceptable due to cultural norms and religious values with an emphasis on virginity…” and also to avoid unwanted pregnancy and STIs. On the other hand with self-administered questionnaires, respondents might have had less inhibition in responding to sensitive issues. Hence the findings that adolescents liked intimate and casual kissing and breast fondling and were more accepting of pre-marital sex (16).

The support of pre-marital sex by fewer participants in our study is linked to the desire of people in some communities to ascertain the fecundity of prospective brides to avoid childlessness. Other reasons provided by male respondents for tolerance of pre-marital sex are based on their misconceptions about abstinence namely; that pre-marital sex is good for girls because it expands the cervix so that child delivery will not be difficult; that without the experience of pre-marital sex, girls will not enjoy sex when married and will experience pains during sexual intercourse, and will not know how to have sex when married; that if boys do not have pre-marital sex, when they get older they will experience reproductive problems. Some of these misconceptions identified in our study reflect the reports of an earlier study in Ogun state, Nigeria (37), which indicated adolescents' perception that abstinence will result in an inability to enjoy sex when married; difficulty in having children as well as painful menstruation.

Mixed responses were obtained about casual sex. While some adolescents regarded casual sex as bad, others felt that it is acceptable. The main reason given by those who reported that casual sex is acceptable is related to their perception that it provides opportunities for girls from poor homes to have the means to interact and enjoy themselves with other adolescents. This finding is consistent with some earlier studies about casual sex in Africa. A study attributed the high incidence of female adolescents' participation in casual sex in Addis Ababa to women's low socioeconomic status (43). Others opined that adolescents' participation in early sex is also partly due to financial constraints among others (38, 44).

The use of transactional sex as a means of survival identified in our study by girls in poverty/economic hardship corroborate findings from some studies in other parts of Nigeria and Africa. In the southwestern part of Nigeria, Folayan reported that more female than male respondents residing more in the rural area engaged in transactional sex (41). This was also attributed to poverty and the desire to meet basic needs. In Zambia, it was reported that girls were willing to engage in sex as long as the boys gave the assurance of providing money, gift or reward in advance, as a strategy for survival but the practice was not regarded as prostitution (45). Also, female students in South Africa engaged in transactional sex (sometimes abusive) due to poverty, with the expectation that material benefits (money, food etc.) provided by men will be exchanged with sexual intimacy (46, 47).

The finding that many girls engage in sex with older men as a means of meeting their basic needs is consistent with other studies (9, 40, 44). The economic benefits of age-disparate sex underscore its interaction with transactional sex. This interaction is corroborated by a study in Tanzania, which reported that a sexual relationship with an older man where money or gifts were not exchanged was perceived by young girls and women as “disgraceful,” “cruel,” “exploitative,” and “dehumanizing” (9). The implication is that age-disparate sex ought to go hand-in-hand with transactional sex, and the reason why young girls engage in sex with older men or young boys engage with sex with older women is that the older adults are more likely to meet their financial needs than their peers of the opposite sex. Furthermore, intergenerational sex was perceived among the participants as immoral and unacceptable based on misconception about age-disparate sex having adverse effects on the physical appearance of the young girl.

This study explored in-depth sexual permissiveness among adolescents, and it highlights some of the reasons for the changing patterns in sexual permissiveness among adolescents in Nigeria. Although we were able to elicit some tolerance for dating and other sexual behaviors from some “bold” adolescents (particularly boys), the use of focus group discussions may have limited the capability of the “less bold” adolescents (particularly the girls) to freely express their true positions in a group. To avoid this limitation in the use of FGDs in discussing sensitive issues, future studies may consider using less invasive interview tools such as one-on-one in-depth interviews or self-administered drop-box questionnaires. It may also be helpful to measure the magnitude of sexual permissiveness among adolescents using quantitative measures. Moreover, we did not seek information on the sexual identity of the FGD participants. This was because sexual identity is not considered a key issue in the discourse on adolescent sexuality currently in Nigeria. This may pose a challenge in generalizing the findings to non-heterosexual youths.

## Conclusion

This paper has identified variations in adolescents' perceptions about dating, pre-marital sex, casual, transactional and age-disparate sex in the study area, which are influenced by socio-cultural and religious beliefs, gender norms, as well as misconceptions about sexuality. Due to the paucity of data on adolescents' perceptions of sexual permissiveness, these findings underscore the need for policymakers and sexual and reproductive health program officers to address the concerns identified such as unwanted pregnancy, unsafe abortion, and exposure to sexually transmitted infections (STIs) and misconceptions about sexuality, which invariably adversely affect the sexual and reproductive health and well-being of adolescents. These call for concerted efforts by policymakers and reproductive health program officers to ensure that adequate sensitization and sexuality education are provided to in-school and out of school adolescents as a strategy for promoting adolescents' access to basic and quality information about sexuality and adolescent sexual and reproductive health (SRH) and debunking misconceptions about sexuality.

Although the Nigerian government introduced a Comprehensive sexuality education (CSE) curriculum in 2002 for schools the literature indicates that many adolescents in Nigeria lack access to reproductive health education programs and youth-friendly services and that there is a tendency for some teachers to withhold SRH information from adolescents due to cultural and religious beliefs. The findings therefore will be invaluable to policymakers and sexual reproductive health program officers in designing and developing intervention strategies that will be appropriate to improve adolescents' sexual health and well-being. Implementation of innovative adolescents' SRH education programs with consideration to the present study findings will be of benefit to this under-served population. The health education programs should target debunking identified misconceptions and equip adolescents with the appropriate knowledge about their sexual health. These could be implemented through media, schools, health facilities including adolescent friendly centers, religious and community forums.

In addition, this work might inform future studies on sexual permissiveness in various ways. A gender and intersectionality study can be undertaken to better understand the gendered perspectives of adolescents and how this interacts with other social stratifiers such as age, schooling status, parental or peer influence, etc. Future studies could also be undertaken in other settings to determine similarities and peculiarities. Finally, since culture is dynamic, we expect changes to occur in adolescents perceptions of sexual permissiveness; so future studies can explore factors contributing to the changes.

## Data Availability Statement

The raw data supporting the conclusions of this article will be made available by the authors, without undue reservation.

## Ethics Statement

The studies involving human participants were reviewed and approved by Health Research Ethics Committee of University of Nigeria Teaching Hospital with Reference Number NHREC/05/01/2008B-FWA00002458-IRB00002323. Written informed consent to participate in this study was provided by the participants' legal guardian/next of kin.

## Author Contributions

CM, NE, and OO conceptualized, designed the study protocol, and data collection instruments. IA, CO, CA, and CM participated in data collection. NE, IA, CO, and CA prepared the first draft of the manuscript. All the authors reviewed and approved the final version of the manuscript for journal submission.

## Conflict of Interest

The authors declare that the research was conducted in the absence of any commercial or financial relationships that could be construed as a potential conflict of interest.
