# Supplementary material for: Adolescents' Perceptions About Dating and Sexual Permissiveness in Ebonyi State, Nigeria: What Can Be Done to Enhance Adolescents' Sexual Health and Well-Being
Source: Front Reprod Health. 2021 Jul 8;3:626931. doi: 10.3389/frph.2021.626931 (PMC9580664; doi:10.3389/frph.2021.626931)
Supplement: Supplementary file 1 [file Presentation_1.pdf]

Addressing unmet need for contraceptives among adolescents using community embedded interventions

FGD guide for adolescents (male and female groups)

Introduction, purpose and procedure

I am a trained data collector from Health Policy Research Group University of Nigeria Enugu Campus and we are working with Ebonyi State government to conduct a study on Adolescent Sexual and Reproductive Health. Adolescent sexual and reproductive health refers to all matters relating to their safe sex life, capability to have children and freedom to decide when to do so. The aim of the study is to address unmet contraceptive need of adolescents in rural and urban areas in Ebonyi State.

We plan to be more involved with your community and engage with young people within the age range of 13 to 18 years over the coming months. We will like to talk with you about your sexual and reproductive health, knowledge/use of contraceptives and ability to access sexual reproductive health services.

Your participation in this discussion is important as it will help us achieve the aim of the study. All information given will be confidential, during and after the research process. Your participation is voluntary and you do not have to answer questions you do not wish to. If you have any question please ask them now or later at the end of the interview.
With your permission, I would like to record this interview to make sure I accurately capture our discussion. This interview will last about 45 minutes.

Ground rules (to be set by moderator and discussants)

Discussion

Sexual Ideology/Gender:

Sexual permissiveness

Probes:

- What do you think about boys and girls dating; hugging/touching; kissing; having sexual intercourse?
- What is the relationship between love and sex?
- What do you think about boys and girls having sex before marriage?
- Is premarital virginity more important/required for girls than boys? Why is this so?
- What do you think about one-night stands or short time among adolescents?
- What do you think about adolescents engaging in sex for money/gifts/favors?
- What do you think about adolescents engaging in sex with older men or women?

Peer influences

Probes:

- Do your peers/friends/school mates approve of one-night stands or 'short-time'? How does this make you feel?
- Do your peers/friends/school mates think that sexual intercourse is alright if a girl and a boy love each other? How does this make you feel?
- What do you think about the number of your peers/friends/school mates who have had sexual intercourse? Did/do you feel pressured by them to have sexual intercourse?
